# Spontaneous transvaginal intestinal evisceration in case of long-standing uterine prolapse

**DOI:** 10.1186/s12893-022-01615-x

**Published:** 2022-05-04

**Authors:** Elena Arabadzhieva, Dimitar Bulanov, Zhivko Shavalov, Atanas Yonkov, Sasho Bonev

**Affiliations:** Department of General Surgery, Unit of Gastrointestinal, Hepato-Biliary and Pancreatic Surgery, University Hospital “Alexandrovska”, Sofia, Medical University of Sofia, 1 G.Sofiiski Str., 1431 Sofia, Bulgaria

**Keywords:** Transvaginal intestinal evisceration, Uterine prolapse, Emergency, Surgical repair, Bowel ischemia, Case report

## Abstract

**Background:**

Transvaginal intestinal evisceration is an extremely rare surgical emergency with potentially fatal consequences. Only a few more than 100 cases with this pathology have been described in the literature. Aetiology is also unclear and multifactoral.

**Case presentation:**

We report the case of an 80-year-old female who presented with sudden severe abdominal pain and spontaneous small bowel evisceration through the vagina along with associated high-grade uterine prolapse. The loops and their mesentery appeared edematous, thickened and dusky, but without apparent necrosis. An urgent laparotomy was performed with subsequent reduction of the prolapsed small bowel into the abdomen, hysterectomy, partial resection of the vagina and vaginal closure. Additional cholecystectomy was necessary because of the visible pathologic changes of the gallbladder. The postoperative period was uneventful. The unique feature of our case is that there was no trigger factor (trauma, constipation or a coughing episode that would increase the intra-abdominal pressure), provoking the vaginal rupture and intestinal evisceration through it in the context of pelvic floor weakness.

**Conclusions:**

Early detection and surgical management are crucial for preventing bowel ischemia and abdominal sepsis. If the eviscerated intestine is ischaemic and non-viable, this requires resection and anastomosis. The approach should be individualized and performed by a multidisciplinary team.

## Background

Transvaginal intestinal evisceration is a rare surgical emergency with potentially fatal consequences [1–6]. A limited number of cases has been published in the literature, so the exact incidence is difficult to determine [7, 8]. Aetiology is also unclear and multifactoral [7, 8]. The usual clinical presentation includes small bowel obstruction due to a herniation of the intestines through the vagina. Diagnosis is made on the imaging tests or during laparotomy [4, 9]. Very rarely, it could present dramatically with large loops of small bowel prolapsing through the vagina resulting in a significant risk of loss of bowel viability. [4, 10]

Our patient presented with sudden severe abdominal pain and spontaneous small bowel evisceration through the vagina along with associated uterine prolapse requiring emergent surgical intervention. This case report is compliant with the CARE Guidelines [11].

## Case presentation

An 80-year-old female living independently presented with sudden severe abdominal pain, weakness, and dizziness. The patient reported she had no constipation or a coughing episode that would increase the intra-abdominal pressure. She did not experience any recent trauma. The patient’s obstetric history included one full-term vaginal delivery and long-standing presence of uterine prolapse (grade 3) treated with pessary placement. However, she stopped using it 2 years ago. Other concomitant diseases were arterial hypertension and anxiety disorder managed with Diazepam. After examination by a gynaecologist, the patient was referred to our department because of established an irreducible small-bowel prolapse through the vagina and suspected changes of the intestinal vitality.

**Fig. 1:**
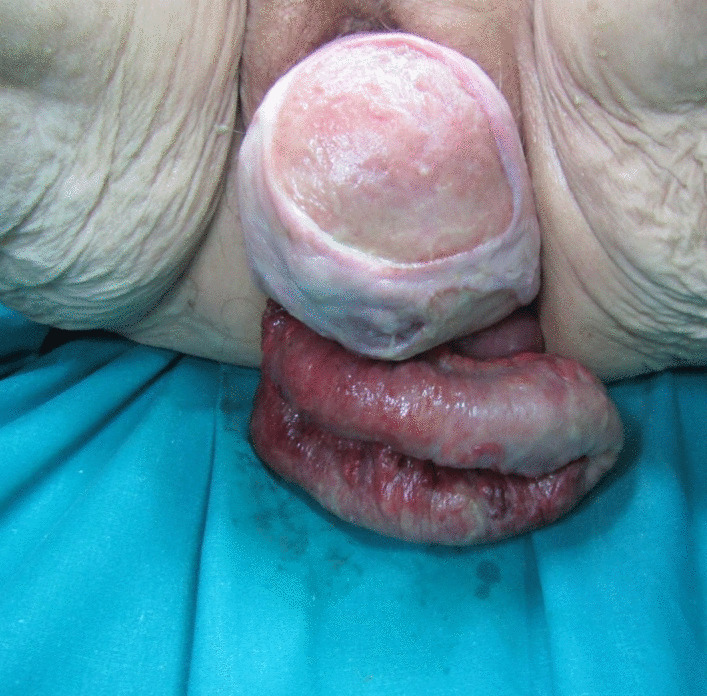
Transvaginal evisceration of the edematous small bowel loops and uterine prolapse at presentation

**Fig. 2:**
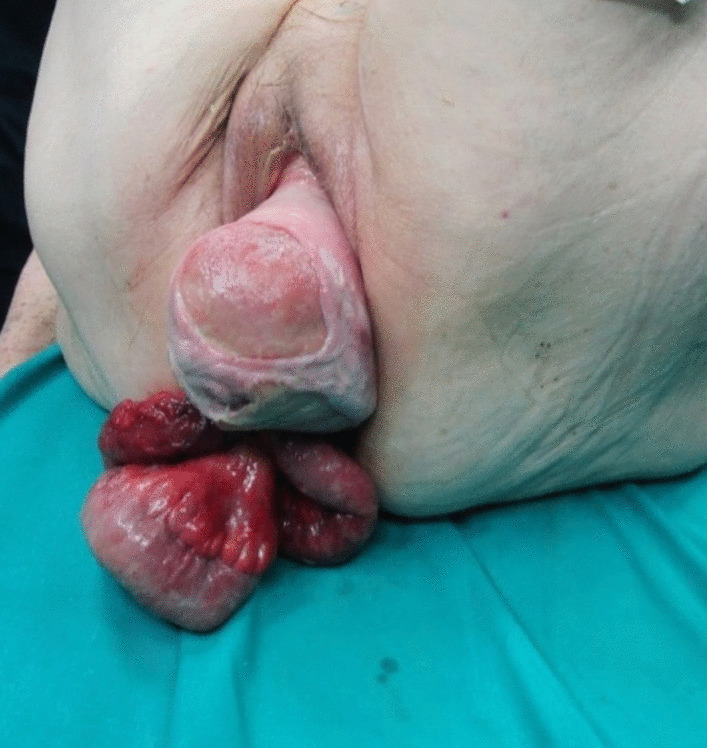
Uterine prolapse and edematous small bowel loops hanging outside the vagina with a view of the dusky appearing mesentery

The physical examination revealed impaired general condition, pale skin, a pulse rate of 90 per minute, and blood pressure of 109/60. The patient’s position in the bed was passive. The abdomen was significantly tender in the lower quadrants with hypoactive bowel sounds. Perineal examination showed 40–50 cm of small bowel prolapsing through the vagina along with associated high-grade uterine prolapse. The loops appeared edematous and thickened, with changed colour, but without obvious necrosis. The mesentery was dusky red and ecchymotic with signs of impaired venous return (Figs. 1 and 2). Routine laboratory tests revealed anaemia (haemoglobin 91 g/l), increased values of leukocytes (12.3 × 109 cells/l), CRP (68 mg/dL), and fibrinogen (6.4 g/l). Blood gas analysis showed decompensated metabolic acidosis (pH 7.24, BE – 9.7, satO_2_ 79%, tCO_2_ 16.5). Fluid resuscitation and intravenous broad-spectrum antibiotic administration (Metronidazole and Cefoperazon) were timely initiated.

Because the intraperitoneal reposition of the eviscerated intestine was not possible and the diagnosis was visible from the examination, CT was not performed to save time. The decision for an early as possible operation was taken into account the status of the prolapsing intestine, and the goal was the preservation of intestinal vitality and avoidance of intestinal resection, if feasible.

The eviscerated intestine was wrapped with warm, sterile, saline-soaked packs. The surgical procedure started with a lower midline incision which later was extended proximally. After assessing the damage, the prolapsed small bowel was reduced into the abdomen. The exploration revealed a laceration of the peritoneum of the Douglas cavity and a defect in the posterior vaginal fornix. The vagina was distended and thickened in its upper third, probably due to the long-term uterine prolapse and pessary use. The affected intestinal loop was localized 30 cm proximally to the ileocecal valve. After the reposition, the small bowel loop restored its vital colour, so bowel resection was not required. Several small mesenteric tears were found and sutured subsequently. The uterus was enlarged due to the presence of several fibroids, and the uterine round ligaments were highly distended. There were no visible signs of malignancies. Intraoperative consultation with a gynaecologist was made because of the changes in the uterus and upper part of the vagina. A hysterectomy with resection of the upper third of the vagina was performed. Vaginal closure, plication of the uterine round ligaments, and fixation to the vaginal cuff were carried out. The peritoneum from the lateral pelvic sidewalls was mobilized, and the repair was performed. Subsequently, the peritoneal cavity was washed with saline. The additional pathological finding was the highly enlarged gallbladder with thickened walls and multiple gallstones inside. So, cholecystectomy was performed. Three drains were inserted—one was placed in the subhepatic space, one over the restored peritoneum of the pelvis, and the last was extraperitoneal, near the vaginal cuff. The same antibiotic regimen initiated prior to surgery was undertaken postoperatively. The postoperative period was uneventful, and the patient was discharged on the 11th postoperative day. Two years later, the patient has no complaints related to the surgery.

## Discussion

Hypernaux et al. described the transvaginal prolapse of abdominal content for the first time in 1864 [12]. Later in 1907, McGregor reported protrusion of small bowel through vaginal wall rupture [13]. Since then, a few more than 100 cases with this pathology have been reported in the literature [1–10, 14]. About 70% of the affected patients are postmenopausal women [3, 6]. This increased incidence could be explained by vaginal wall atrophy, which coincided with the triad of hypoestrogenism, chronic tissue devascularization, and pelvic floor weakness [10, 15]. Most patients with transvaginal evisceration have previous gynaecological surgery or concomitant pelvic organ prolapse [10, 15, 16]. According to a review of all hysterectomies and pelvic repairs performed at Mayo Clinic from 1970 through 2001, Croak et al. reported a 0.032% incidence of vaginal evisceration after a pelvic operation [10]. Somkuti et al. described ten risk factors for apical vaginal rupture after an abdominal or vaginal hysterectomy: (1) poor technique, (2) postoperative infection, (3) hematoma, (4) coitus before healing, (5) age, (6) radiotherapy, (7) corticosteroid therapy, (8) trauma or rape, (9) the previous vaginoplasty, and (10) use of the Valsalva manoeuvre [17]. Many other factors can influence this condition, such as lifestyle, hypothyroidism, obesity, multiparous women, previous pelvic radiotherapy, and poor collagen structure [1–10]. However, most case reports on the topic described a trigger moment for the evisceration as recent trauma or surgery, coughing, constipation or any other factor that would increase the intra-abdominal pressure suddenly in the context of pelvic floor weakness [8, 16, 18, 19]. In premenopausal women, transvaginal intestinal evisceration is extremely rare and often associated with instrumentation, obstetric injury or coital trauma, which vaginal lacerations may accompany [7, 10, 14, 20]. Our patient was a postmenopausal woman with concomitant uterine prolapse, which may have weakened the vaginal wall. However, the unique feature of the case is that there was no event provoking the vaginal rupture and intestinal evisceration through it.

Transvaginal small bowel evisceration is related to 6–8% mortality and a high morbidity rate (15–20%) [1, 3, 4, 21]. Complications associated with this condition include intestinal ischaemia and gangrene, abdominal sepsis and deep vein thrombosis [1, 2, 22]. Because of that, early recognition and surgical treatment are crucial. Due to the rarity of this condition, there is no unified consensus about the optimal surgical technique [23]. So, the surgical approach should be individualized and performed by a multidisciplinary team. Guttman and Afilalo emphasized five key points that may aid in the acute management of rupture and evisceration: (1) stabilizing the patient; (2) managing the patient’s fluid status, especially in patients with shock; (3) preserving the bowel in a moist saline wrap; (4) administering broadspectrum antibiotics to cover gastrointestinal flora, and (5) initiating the immediate surgical repair [10]. The transvaginal intestinal evisceration management must start with a detailed assessment of the herniated viscus. A reduction may be attempted if the eviscerated bowel is viable, has not been previously irradiated, and there are no signs of an acute abdomen. Subsequent transvaginal surgical repair may be feasible [3, 4]. This, however, limits thorough inspection of the bowel length [19]. So, in these cases, a combined laparoscopic and vaginal approach may be beneficial to enable appropriate inspection of the abdominopelvic viscera before repairing the vaginal defect [8, 19]. However, in most cases such as ours, the intraperitoneal reposition of the eviscerated intestine was not possible (due to the status of the affected loops or the vaginal defect is located too high) [19]. So, this required laparotomy combined with a transabdominal or transvaginal vault repair [3, 4, 10, 19]. Ischaemic, non-viable bowel would require resection and anastomosis [3–5, 7, 10]. In our case, after transabdominal reduction, eviscerated small intestine restored its vital colour and hysterectomy, partial resection of the vagina and vaginal closure was performed due to high-grade uterine prolapse. Additional cholecystectomy was necessary because of the visible pathologic changes of the gallbladder.

## Conclusions

Transvaginal intestinal evisceration is an extremely rare emergency. Early detection and surgical management are crucial for preventing bowel ischemia and abdominal sepsis. If the eviscerated intestine is ischaemic and non-viable, this requires resection and anastomosis. The approach should be individualized and performed by a multidisciplinary team.

## Data Availability

The data sets supporting the conclusions of this article are included in the report.

## Abbreviations

CT: Computed tomography
CRP: C-reactive protein
satO_2_: Oxygen saturation
BE: Base excess
tCO_2_: Total carbon dioxide
cm: Centimetres
